# Autism Spectrum Disorder and miRNA: An Overview of Experimental Models

**DOI:** 10.3390/brainsci9100265

**Published:** 2019-10-03

**Authors:** Giovanni Schepici, Eugenio Cavalli, Placido Bramanti, Emanuela Mazzon

**Affiliations:** IRCCS Centro Neurolesi “Bonino-Pulejo”, Via Provinciale Palermo, Contrada Casazza, 98124 Messina, Italy; giovanni.schepici@irccsme.it (G.S.); eugenio.cavalli@irccsme.it (E.C.); placido.bramanti@irccsme.it (P.B.)

**Keywords:** autism spectrum disorder, miRNA, experimental models

## Abstract

Autism spectrum disorder (ASD) is a complex neuropsychiatric disorder characterized by deficits in social interactions, communication, language, and in a limited repertoire of activities and interests. The etiology of ASD is very complex. Genetic, epigenetic, and environmental factors contribute to the onset of ASD. Researchers have shown that microRNAs (miRNAs) could be one of the possible causes associated with ASD. miRNAs are small noncoding mRNAs that regulate gene expression, and they are often linked to biological processes and implicated in neurodevelopment. This review aims to provide an overview of the animal models and the role of the different miRNAs involved in ASD. Therefore, the use of animal models that reproduce the ASD and the identification of miRNAs could be a useful predictive tool to study this disorder.

## 1. Introduction

Autism spectrum disorder (ASD) affects about 2% of the world population, with a major incidence for males compared to females. Patients with ASD show abnormal, repetitive, and stereotyped behaviors; these abnormalities involve deficits related to cognitive functioning, learning, attention, and sensory development. ASD symptoms coexist with other psychiatric and medical conditions such as intellectual disability, epilepsy, deficit in motor control, attention deficit hyperactivity disorder, gastrointestinal problems, tics, sleep disorders, and anxiety [1]. The meta-analysis of the genes involved in ASD has shown their involvement in numerous biological pathways such as protein synthesis and degradation, dynamics and cytoskeletal structure, synaptic function, and regulation of gene expression. Studies on ASD pathogenesis have shown miRNAs have a direct role in the ASD onset [2,3]. miRNAs are implicated in different cellular processes, such as development, proliferation, differentiation, growth control, homeostasis, and apoptosis. They constitute a class of small noncoding RNAs composed of 20–22 nucleotides. miRNAs are post-transcriptional regulators; they bind to the 3’ untranslated region (UTR) of messenger RNAs (mRNAs) in order to inhibit the translation or degradation of mRNA. The biogenesis of miRNAs occurs through several steps. In the nucleus, miRNA is transcribed by RNA polymerase II or III into a hairpin structure called primary miRNAs (pri-miRNA). Subsequently, about 25% of cluster-organized miRNAs are transcribed into a single polycistronic pri-miRNA. Therefore, the pri-miRNA is cleaved from the Drosha and DGCR8 complex in precursor miRNAs (pre-miRNA). The pre-miRNA is translocated by Exportin-5 (XPO5), into the cytoplasm. In the cytoplasm, the pre-miRNAs are split by Dicer RNAase III, creating dissociate duplexes of miRNAs that are potentially mature. One of the two strands associated with Argonaute proteins (AGO 1–4) is called mature miRNA and is composed of 22 nucleotides. Dicer and TAR-RNA binding protein (TRBP) form the RNA-induced silencing complex (RISC) that leads to the target mRNA. Only one filament is incorporated in RISC, while the other is degraded. The RISC–miRNA complex binds the target mRNA and induces or inhibits the translation or degradation of mRNA transcription. Since in most cases, imperfect coupling occurs, as a consequence, there is the translational repression of the target protein [4,5,6]. Reduced levels of miRNAs expressed in the brain, blood, saliva, and other body fluids have been observed in patients with ASD, making them essential biomarkers for ASD identification [7]. This review treats the role of miRNAs in the pathogenesis of ASD. Furthermore, it investigates the animal models of ASD to deepen our knowledge of this type of disorder.

## 2. ASD in an Experimental Model

Several factors contribute to the onset of ASD. Genetic association studies have shown how mutations in some genes can determine the onset of ASD phenotypes, including Fragile X mental retardation 1 (FMR1), methyl-CpG binding protein gene of type 2 (MECP2), Neuroligin (NLGN), Neurexin 1 (NRXN), Tuberous sclerosis 1 and 2 genes (TSC1 and TSC2), Reelin (RELN), Engrailed2 (EN2), and Phosphatase and tensin homolog protein (PTEN). The identification of these genes has led researchers to study animal models that reproduce ASD. Therefore, the use of transgenic mouse models has proven to be a very useful tool to study the pathophysiological mechanisms of ASD and discover new potential therapeutic treatment [8].

The *FMR1* Knock-Out (*FMR1*-KO) is an animal model induced by gene targeting that involves the deletion of exon 5. Researchers have shown that the deletion of the *FMR1* gene causes a mental delay in mice as it does in human beings. The *FMR1*-KO mouse model has shown abnormalities in the development of dendritic spines and pyramidal cells in the cerebral cortex which are involved in neuroplasticity. In addition, this mouse model showed a decrease in social interactions and in the fear of danger as seen in human FXS and ASD [8,9].

Rett syndrome (RTT) is a complex neurological disorder caused by the deletion of *MECP2*. This gene is a transcriptional repressor and is located on the X chromosome. This deletion involves loss of function, determining the RTT. A useful animal model for studying RTT is the *MECP2*-KO mouse model. Two different *MECP2*-KO models were generated. One model involves the deletion of exon 3–4, while the other one generated by removal of the entire exon 3 and part of exon 4 and introduction of the nonsense sequence [10,11]. The most severe form of RTT has been observed in *MECP2*-KO male mice, reporting lethality at 10 weeks of age [12]. In addition, *MECP2* Knock-In (*MECP2*-KI) was generated through the addition of missense mutations in which threonine 158 is converted to alanine [13]. 

NLGN and NRX are proteins encoded by *NLGN* and *NRX* genes. These proteins are involved in cell adhesion, neurotransmission, and synaptic differentiation. Mutations in *NLGN* and *NRX* genes may involve behavioral changes and social interactions such as in ASD [14]. On the basis of these mutated genes, some mouse models have been developed [15]. The *NLGN1*-KO mouse model was induced by the deletion of the first two coding exons. Studies conducted on *NLGN1*-KO mice have shown deficits in behavioral tests related to anxiety and spatial learning. Furthermore, these animal models have shown an alteration to the stimuli induced by heat and pain in tests. Similarly, these deficits have been observed in patients with ASD [16]. 

*NLGN2* is a gene that codes for the postsynaptic cell adhesion protein NLG2. This protein supports the integrity and functionality of inhibitory synapses and it is also involved in neuropsychiatric and depressive diseases [17]. The *NLGN2*-KO mouse model involves the deletion of the first coding exon. Studies conducted in the prefrontal cortex and in the hippocampus of *NLGN2*-KO mice confirmed the role of NLGN2 in maintaining the function of inhibitory synapses [18]. In addition, behavioral studies have shown an increase in anxiety, alterations in the perception of pain-induced stimuli, and a decrease in motor coordination. By contrast, they have shown social behavior interactions and a normal locomotor activity [19].

The *NLG3*-KO mouse model involves the deletion of exons 2–3; this mouse model has shown the alteration of synaptic plasticity correlated to a mild ASD phenotype. Further, researchers have observed a regular social interaction but an increasing of anxiety in behavior tests [20].

*NGL3*-KI mice were induced by basis replacement of cysteine with arginine in *NLGN3* located on chromosome X. Studies conducted on *NLG3*-KI mice showed a reduction in sociability and an improvement in spatial learning compared to *NLG3*-KO [21,22]. In addition, Chadman et al. [23], in a study performed on *NLG3*-KI mice, observed developmental delays and deficits in motor learning. 

NRXN1 is a protein present on the presynaptic cell surface and biologically linked to postsynaptic NLGN. In mammals, NRXN1 is encoded by the *NRXN1* gene. Further, *NRXN1* has two isoforms, Neuroxine α (*NRXα*) and Neuroxine β (*NRXN*β) [24]. Studies on mice have shown that mutations in the *NRXN1* gene are associated with ASD, schizophrenia, and other neuropsychiatric disorders. The *NRXN1α*-KO mouse model as a result of the deletion of *NRXN-1α* has demonstrated a mild phenotype associated with ASD. Only in females mouse, the researchers have observed hypoactivity. Instead, an increase in aggressive and anxiety-related behaviors has been observed in males. Furthermore, sex-related behavioral alteration in mice is one characteristic of ASD patients; hence, these models are useful for further research on this type of disorder [25].

The *SHANK* genes encode Src Homology-3 (SH3) and multiple ankyrin repeat domains proteins (SHANKs). Mutations in *SHANK* includes three genes (*SHANK* 1–3) related to ASD. The deletion of *SHANK3* located on chromosome 22 determines the Phelan–McDermid syndrome, characterized by autistic phenotypes that leads to a linguistic deficit in intellectual and motor development [26]. To better understand the role of these genes in ASD, genetic mutations have been reproduced in mouse models. Studies using *SHANK1*-KO mice, obtained by the deletion of exons 14 and 15, showed an increase in anxiety, hypoactivity, deficits in motor learning, and conditioning to fear [27]. Researchers employed two types of *SHANK2*-KO mice, one with deletion of exons 6–7 and the other one with deletion of exon 7. Won et al. [28], using the *SHANK2*-KO mice model with deletion of exons 6–7, showed in behavioral tests a decrease in interaction and social communication, memory deficits and spatial learning, hyperactivity, and an increase in anxiety-related behavior. Schmeisser et al. [29], reported behavioral alterations in tests such as increased self-cleaning, anxiety, and hyperactivity, exploiting the *SHANK2*-KO mice model with deletion of the exon 7. Several *SHANK3*-KO mice have been employed to demonstrate the ASD phenotype. Bozdagi et al. [30], using *SHANK3*-KO mice with a deletion of exons 4–9, observed normal interest sociability in behavioral tests in mouse puppies. In addition, slight hypoactivity in the open field and in interactions between young male-female couples were observed. Moreover, Yang et al. [31], using *SHANK3*-KO mice, showed the alteration of motor performance and deficits in recognition of new objects and in learning in tests [30,31]. Wang et al. [32], using *SHANK3*-KO mice with deletion of exons 4–9, showed alteration of social interaction. Furthermore, deficits in food preferences and motor performance, while normal levels of anxiety have been observed.

Peça et al. [33], using two types of the *SHANK3*-KO mice model, one with deletion of exons 4–7 and another with a deletion of exons 13–16, have found mostly the ASD phenotype in *SHANK3*-KO mice with the deletion of exons 13–16. These animals showed a decrease in sociality and male-female interaction. In addition, they showed an excessive increase in anxiety and self-cleaning, with skin lesions often occurring. 

Kouser et al. [34], exploiting the *SHANK3*-KO mice model characterized by the deletion of exon 21, reported normal social interaction and a complicated nesting in mice. Furthermore, an increase in anxiety and learning and memory deficits in tests was observed.

*TSC1* and *TSC2* are genes of considerable interest. These genes are located, respectively, on chromosomes 9 and 16, encoding for the proteins Hamartin and Tuberin. Mutations in one of the two *TSC* genes determine the onset of tuberous sclerosis, an autosomal neurodevelopmental disorder with autistic spectrum symptoms [35]. *TSC* mutant mouse models have been chosen to deepen the knowledge of this ASD phenotype. The researchers employed two *TSC1* mice models, one with deletion of exons 6–8 and the other with deletion of exons 5–7 to generate the nonfunctional copy of this gene. The results of these studies showed how *TSC1* mutations in homozygous mice are lethal. By contrast, heterozygous *TSC1* mice showed ASD behaviors as a poor male-female interaction within the couple and a compromised nest construction [36]. Tsai et al. [37], in a study conducted on the *TSC1*-KO mice model, observed a significant increase in size and a decrease in the excitability of the Purkinje cells. In behavioral tests, a deficit of social interaction and an increase in repetitive behaviors were shown.

Reith et al. [38], using the *TSC2*-KO mouse model, observed lethality in homozygous mice, while heterozygote mice showed an ASD-related phenotype. Only females’ deficits in social behavior were reported, while the males showed little interest in social novelty and in male-female interaction. Furthermore, deficits in motor performance, learning, and memory were observed. 

REEL is a protein involved in neurodevelopment, encoding by *RELN*, a gene located on chromosome 7. The *RELN* role is to regulate dendritic budding in GABAergic cerebellar Purkinje cells [39,40]. A study performed by Hadj-Sahraoui et al. [41] showed a reduction of Purkinje cells in the cerebellum of *RELN* heterozygous mutant mice in a period between 3 and 16 months of age. This impairment was observed only in males mice. By contrast, in female mice, no deficits were observed in Purkinje cells. Therefore, these results suggested that *RELN* exerts its gender-specific action, as demonstrated by the increased incidence of ASD in males [40].

Related to these previous studies is the *EN2* gene. This gene is located on chromosome 7 and encoding for the EN2 protein involved in embryonic development of the midbrain and central nervous system. Studies on the *EN2*-KO mice model showed a decrease in Purkinje cells. Furthermore, researchers demonstrated a reduction in the size of the hippocampus and an ectopic disposition of neurons [42]. Investigations in *EN2*-KO mice showed behavioral abnormalities such as deficits in social behavior and memory. Therefore, *EN2*-KO mice showed evidence of morphology and similar behaviors with ASD patients [43].

Another gene of considerable interest is *PTEN.* This gene is a gene located on chromosome 10 and encodes for the PTEN protein involved in the regulation of the cell cycle. *PTEN* is important in synaptic plasticity, in neuronal function, and development. *PTEN* mutations have been associated with ASD phenotypes such as the Cowden, Bannayan–Riley and Proteus syndromes [44]. *PTEN*-KO mice models are a useful means for planning the study of ASD behaviors. Kwon et al. [45], using the *PTEN*-KO mice model, showed macrocephaly and also loss of neuronal polarity in the hippocampus and in the cortex. Furthermore, some behavioral anomalies associated with ASD such as anxiety, convulsions, and decreased social interest were experienced in these animals.

It has been observed that women with mutations in the 15q11-q13 gene locus of chromosome 15 determine progeny affected by the Angelman syndrome. Furthermore, it has been observed that the duplication or triplication in locus 15q11-q13 of maternal origin is the most common cytogenetic anomaly associated with ASD. The ubiquitin-protein ligase E3A protein encoded by the *UBE3A* gene that is located on locus 15q11-q13 was observed. *UBE3A* plays an important regulatory role in the development of neural circuits and mammalian synaptic plasticity [46]. *UBE3A* mutations are associated with Angelman syndrome, a disorder characterized by severe somatic and intellectual developmental delay, deficits in speech development, sleep disorders, and motor dysfunction [47]. The transgenic mice model induced by the duplication of *UBE3A* has proven very useful in better understanding this disorder. Behavioral tests conducted on *UBE3A* mutant mice have shown a reduction in sociability and an increase in self-care [48]. Table 1 summarizes the list of knockout mouse models related to autism spectrum disorders.

Researchers also used nongenetic animal models to study ASD behaviors. A mouse model that was used is that induced by compounds that determine maternal immune activation (MIA) during gestation. C57BL/6 mice were exposed to polyinosinic:polycytidylic acid (Poly I:C) during pregnancy to mimic viral and bacterial infection. The progeny resulting from (Poly I:C) exposure showed different immunological, neurological, and behavioral deficits such as in ASD. Researchers found little sociability in the behavioral tests [49]. Another animal model of ASD used prenatal exposure to valproic acid (VPA), an antiepileptic drug that stabilizes mood. This model is a useful tool for investigating the etiology and biology of ASD [50]. The researchers showed how, in rodents, prenatal exposure to VPA could induce behavioral deficits such as those seen in ASD. Therefore, based on these observations, in 1996, the first model was created by administering a single dose of 350 mg/kg of VPA to rat embryos [51]. ASD can be defined only by behavioral observations. Male rats showed numerous behaviors associated with ASD. The rats showed an increase in repetitive and compulsive behaviors and a reduction in sociality, attention, and spinal sensitivity induced by pain. In addition, the researchers observed deficits in olfactory discrimination, reduction in body weight, and delays in motor development [52]. Behavioral tests evaluating interaction and social communication were performed. Furthermore, the labyrinth test was used to observe repetitive behaviors. Anxiety levels were assessed by measuring the time spent with open arms in the labyrinth. Further tests regarding food preferences showed how VPA rats tend to eat less tasty foods compared to the control rat [53]. Based on these observations, the VPA rodent model can be classified as useful for the study of neurobiology and the behavior of ASD [54].

## 3. miRNA and ASD 

Underlying the pathogenesis of ASD, recent studies have shown that miRNAs are involved in the development of the examined disorder. The mouse models, designed after the discovery of the mutated gene responsible for the ASD form, proved to be useful tools for researchers to identify the causes of the disease. 

Edbauer et al., using *FMR1*-KO mice, demonstrated that the silencing of the *FMR1* gene encoding for the Fragile X mental retardation protein (FMRP) determines the onset of FXS. The researchers observed that miRNA-125b and miRNA-132 are important in the regulation of FMRP translation. In addition, these miRNAs act differently on the morphology of the spine and synaptic plasticity in the hippocampal neurons. The identification of miRNAs in the brain of these mice was performed by extracting the FMRP protein and by marking it with specific antibodies to identify the mRNA targets of the protein. The subsequent quantitative analysis allowed identifying 12 miRNAs with FMRP as a target. Moreover, the overexpression of miRNA-125b and miRNA-132 in hippocampal neurons after plasmid transfection was evaluated. miRNA-125b upregulation caused the development of long and thin spines, while miRNA-132 upregulation led to an increase in the average width of the spines and reduced spinal density. miRNA-125b upregulation led to a reduction in synaptic transmission, as demonstrated by long and thin spines. By contrast, miRNA-132 upregulation led to an increase in synaptic frequency, as confirmed by the width of the spines [55]. In addition, it was also demonstrated that miRNA-125b targets the NR2A subunit of the N-methyl-D-aspartate receptor (NDMAR) that influences synaptic plasticity and memory consolidation. Therefore, the study of the NDMAR function in these mice could lead to new strategies for the treatment of FXS.

Zongaro et al., using *FMR1*-KI mice with tremor/ataxia syndromes associated with Fragile X (FXTAS), observed behaviors associated with ASD. FXTAS is a more severe form of FXS, in which the overexpression of *FMR1* that occurs in adulthood causes developmental and behavioral deficits. In tests of the aquatic labyrinth, these mice aged between 52 and 72 weeks showed numerous cognitive deficits. The researchers, using luciferase activity, which binds the 3’UTR sequence of the *FMR1* gene, showed how miRNA-101, miRNA129-5p, and miRNA-221 regulate the expression of *FMR1* in the brain. In addition, the synaptic expression levels of miRNA-129-5p, miRNA-221, and miRNA-101 were compared with miRNA-134 levels. The expression of miRNA-221 was not detectable during embryonic life. While the authors reported that miRNA-129-5p expression was increased in the postnatal period, miRNA-221 was increased in the adult phase. In addition, the study of Zongaro et al. reported that miRNA-221 upregulation determines loss *MECP2* function such as in RTT [56].

The results on KI mice showed that overexpression of *FMR1* causes synaptopathy, a characteristic of phenotypes of ASD, such as in FXSTAS. In addition, in the study of Zongaro et al., it was reported that miRNA-221 upregulation determines the loss of *MECP2* function such as in RTT. 

Urdinguio et al., using *MECP2*-KO mice, observed the dysregulation of some miRNAs. The subsequent brain transcriptomic analysis showed that miRNA-146a, miRNA-146b, miRNA-130, miRNA-122a, miRNA-342, and miRNA-409 were downregulated, while miRNA-29b, miRNA-329, miRNA-199b, miRNA-382, miRNA-296, miRNA-221, and miRNA-92 were upregulated. Urdinguio et al. demonstrated that miRNA-146a and miRNA-146b are involved in ASD etiopathogenesis. miRNA-146a and miRNA-146b targeted the 3’-UTR sequence of interleukin-1 receptor-associated kinase 1 (IRAK1) encoded by the *IRAK1* gene. The IRAK1 protein, engaging with one of the two serine/threonine kinases, binds the interleukin-1 triggering inflammatory function [57]. The researchers subsequently evaluated the regulatory role of miRNA-146a and miRNA-146b in a neuronal context, transfecting them into murine neuroblastoma cells [58]. They showed that in healthy brain conditions, miRNA-146a and miRNA-146b determine the down-expression of *IRAK1*. In addition, the downregulation of miRNA-146a and miRNA-146b causes *IRAK1* overexpression by inducing an inflammatory state of brain tissue and by contributing to the phenotype RTT [59]. 

Zhang et al., using *MECP2* transgenic mice, showed how this gene is implicated in the pain pathway by acting as an analgesic mediator. The study proved deficits in social interaction, anxiety, and motor functions. The researchers observed that *MECP2* overexpression is linked via the p-CREB/miR-132 protein pathway. This complex, binding to the cAMP, generates the reduction of pain induced in these mice. miRNA-132 targets the 3′UTR sequence of *MECP2* when it is upregulated and inhibits the level expression at the post-transcriptional level of *MECP2*. The downregulation of miRNA-132 leads to an increase in *MECP2* expression related to increased analgesic response to mechanical and thermal stimulation [60].

Lyu et al., showed in an in vitro study using primary mouse neurons, that miRNA-137 and miRNA-132 regulate *MECP2* and *PTEN* expression. The *MECP2* knockout upregulated miRNA-137, which in turn leads to *PTEN* down-expression. Furthermore, *PTEN* knockout was observed to upregulate miRNA-132. Therefore, the study showed how the genes *MECP2* and *PTEN* regulate their expression through miRNAs [61].

Cheng et al. characterized a new KO mouse model for *MIR137.* They observed how this gene is essential for mouse postnatal development, which is important as the loss of function of *MIR137* has been observed in ASD. Furthermore, they demonstrated that the partial loss of miRNA-137 function causes alterations in synaptic plasticity and behavior [62]. 

Cortabitarte et al. performed in silico and in vitro studies on the *SHANK* genes, which code for postsynaptic scaffold proteins. In the in silico study, they identified a single miRNA-137 binding site in the *SHANK*2 gene. In the in vitro study, on mouse hippocampal neurons, they showed through Western blot analysis how the upregulation of miRNA-137 reduces the *SHANK2* expression levels. Therefore, the overexpression of miRNA-137 involves the downregulation of the SHANK2, an important protein involved in the development of the postsynaptic structure [63]. 

*UBE3A* plays an important role in neural development and synaptic plasticity. Vatsa et al., using the Angelman syndrome mouse model, showed how miRNA-708 has been downregulated. In addition, they observed that miRNA-708 targets the *NNAT* gene that encodes for Neuronatin protein (NNAT). This gene is involved in the regulation of ion channels during brain development. The researchers observed that downregulation of miRNA-708 leads to an increase in intracellular Ca2⁺. Moreover, they observed the phosphorylation of Calcium/calmodulin-dependent protein kinase type II alpha chain (CAMKIIα), encoded by the *CAMK2A* gene. CAMKIIα protein is involved in synaptic plasticity. In addition, an increase was observed in NNAT levels and GABAergic neurons. The results of the study showed how miRNA-708 acts on *NNAT* mediating the aberrant Ca2⁺ signaling, thus contributing to the pathogenesis of the Angelman syndrome [64].

Valluy et al., using the *UBE3A*-KO mouse model, demonstrated that *UBE3A1* transcription encodes an inactive UBE3A1 protein. The UBE3A1 protein, as it lacks the E3 ligase responsible for catalytic activity, prevents dendritic growth and promotes the maturation of the dendritic spine in these mice. The study showed how the miR379-410 cluster in particular miRNA-134 competes with UBE3A1 through the 3’-UTR sequence for the mRNA target. During neural development, it was observed that both the down-expression of *UBE3A1* and the upregulation of the miR379-410 cluster containing miRNA-134 favored the dendritic growth but not that of spine maturation. Therefore, the dysregulation of the miR379-410 cluster and miRNA-134 can determine the onset of deficits in neurodevelopment as in ASD [46]. 

Lackinger et al., using the miR379-410-KO mouse model, observed the correlation between loss of miR379–410 functions and alteration in social behavior. MiR379–410 is a cluster located on chromosome 12 in mice. The KO of the miR379-410-KO mouse model showed an increase in excitatory synaptic transmission in the hippocampus, resulting in altered social behavior and increased anxiety. Furthermore, no memory deficits were observed in social and object recognition tests. In conclusion, this study showed that the miR379-410 cluster influences the development of social behaviors in mice as in patients with ASD [65].

Hirsch et al. evaluated the effects of treatment with resveratrol (RSV), an antioxidant and anti-inflammatory molecule, in a rat model of ASD induced by the administration of VPA. The study showed that RSV prevents behavioral deficits in VPA rats. Furthermore, the expression of circulating miRNAs in VPA rats was estimated before and after the treatment with RSV. An increase in the miRNA-134-5p level was identified, while treatment with RSV prevented the alteration of these miRNA levels. Furthermore, the expression of miRNAs of patients with ASDs compared to healthy patients was evaluated. The results showed an increase in the levels of miRNA134-5p and miRNA138-5p in patients with ASD [66].

miRNA-134-5p and miRNA-138-5p acted in the brain, dynamically regulating the cytoskeleton through two antagonistic pathways. miRNA-134-5p could negatively affect the size of dendritic spines in postsynaptic sites and inhibit the translation of the protein domain kinase 1 (LIMK1) encoded by the *LIMK1* gene. By contrast, miRNA-138-5p is involved in the development of dendritic spines. miRNA-138-5p regulates the expression of the acyl thioesterase 1 (APT1) protein encoded by the *APT1* gene. APT1 is an enzyme that regulates the depalmitoylation of the Gα protein and, consequently, the morphogenesis of the dendritic spine. The results of the study demonstrate an important mechanism of control on vertebral column morphogenesis exerted by miRNA-138-5p on *APT1* expression [67].

Dai et al., using the VPA-rat model, shown that miRNA-34a regulates the expression of *BCL2*, although the mechanisms are not entirely known. They observed in the cerebral cortex of VPA rats, immediately after birth, the upregulation of miRNA-34a and *BCL2* down-expression. Consequently, the miRNA-34a/Bcl-2 signaling pathway could be an important way to activate ASD in VPA rats [68]. Moreover, the exposure to VPA in rats caused alterations in the miRNA-181c and miRNA-30d profiles. Furthermore, these animals produced an enlarged amygdala, thus confirming the behavioral alterations observed also in ASD patients. The amygdala transcriptomic analysis showed alteration of the neuronal morphology that can be traced to a post-transcriptional process regulated by miRNAs. The results of these analyses suggested that high levels of miRNA-181c and miRNA-30d can provide a dysregulated expression on their mRNA targets that encode proteins involved in neuronal development and that contribute to the pathophysiology of ASD [69,70]. 

Schneider et al., in a study on the amygdala of VPA rats, evaluated miRNA expression and studied the behaviors. Subsequent analyses showed the upregulation of miRNA-181c and miRNA-30d in VPA rats. Further, analysis of the genes containing a binding site for miRNA-30 and miRNA-181c was performed. miRNA-30d targets numerous genes whose function is mainly associated with the development of the nervous system. The researchers also observed motor deficits and an increase in anxiety in behavioral tests. In conclusion, the dysregulation of these miRNAs can be considered an important event for neurodevelopment, determining the onset of characteristics similar to ASD as evidenced in the VPA rat model [71]. Table 2 summarizes all miRNA correlated to autism spectrum disorders with their functions and relative mice model.

## 4. Conclusions

Research has revealed that ASD has a genetic basis, as well as epigenetic and environmental factors may contribute to its development. Based on these observations, researchers planned animal models capable of reproducing and studying the ASD phenotype. Animal models are an important means of linking specific changes in miRNA biogenesis and the molecular role they play in the etiopathogenesis of neuropathological and behavioral abnormalities. Murine models have been reproduced based on mutations found in patients with ASD. Additionally, nongenetic ASD animal models obtained from prenatal exposure to VPA or induced by immune activation following infection have been useful for researchers to study this disorder. In recent years, miRNAs have attracted the attention of researchers by showing promising results and by signifying that these molecules have a straight role in the pathogenesis of ASD. Research showed that several miRNAs, including miRNA-125b, miRNA-132, miRNA-146a, miRNA-146b, miRNA-137, miRNA-134, miRNA-134-5p, miRNA-138-5p, miRNA-34a, miRNA-181c, and miRNA-30d, are directly linked to ASD. Therefore, since miRNAs regulate the expression of several genes involved in neurodevelopment, they are directly involved in ASD. Furthermore, on the basis of these observations, researchers have identified miRNAs as an important tool to identify new therapies in order to obtain the best and supportive results for ASD. Moreover, miRNAs can be identified as potential biomarkers and validated for the diagnosis and prognosis of the autistic spectrum or can even be used to distinguish different phenotypes of a specific psychiatric disorder, such as ASD.

## Figures and Tables

**Table 1:** Genes associated with autism spectrum disorder (ASD) for which a Knock-Out model is provided. The column variants are “*AutDB variants*”, including the variants in the protein-coding region that is AutDB-related and with a familial origin.

Human Gene	Locus	Aut DB Variants	Associated Disease	Model
***FMR1***	Xq27.3	p.Lys547Glu	Fragile X syndrome	*FMR1*-KO
		p.Glu360Gly		
		p.Ser27Ter		
		p.Arg138Gln		
		p.Ile580fsTer9		
		p.Gly266Glu		
		p.Met140IlefsTer3		
***MECP2***	Xq28	p.Glu406Ter	Rett syndrome	*MECP2*-KO
		p.Ala140Val		
		p.Arg167Trp		
		p.Pro388_Pro467del		
		p.Pro322Ser		
		p.Ala214Gly		
		p.Thr240Ser		
		p.Ala370Thr		
		p.Pro199Arg		
		p.Glu483Ter		
		p.Glu318Asp		
		p.Val320His		
		p.Arg354_Val412delins41		
		p.Glu495Ter		
		p.Gly185Val		
		p.Arg167Trp		
		p.Arg309Trp		
		p.Ala140Val		
		p.Pro398fsTer		
		p.Glu483Ter		
		p.Glu495Ter		
		p.Arg190His		
		p.Arg133Cys		
		p.Arg306Cys		
		p.Arg133Cys		
		p.Pro389Ter		
		p.Arg470His		
		p.Asp147Glu		
		p.Pro376Ser		
		p.Gly249fs		
		p.Val380CysfsTer27		
		p.Pro403Arg		
		p.Glu406Lys		
***NLGN3***	Xq13.1	p.Arg451Cys		*NLGN3*-KO
		p.Thr632Ala		
		p.Val321Ala		
		p.Val306Met		
		p.Trp463Ter		
		p.Arg617Trp		
		p.Thr429fs		
		p.Thr449fs		
***NLGN4X***	Xp22.32–p22.31	p.Lys378Arg		
		p.Gln329Ter		
		p.Arg766Gln		
		p.Gly84Arg		
		p.Ala283Thr		
		p.Gly99Ser		
		p.Lys378Arg		
		p.Val403Met		
		p.Arg704Cys		
		p.Glu418AspfsTer12		
		p.Gln274Ter		
***TSC1***	9q34.13	p.His732Tyr	Tuberous sclerosis	
		p.Lys587Arg		
		p.Ser403Leu		
		p.Thr360Asn		
		p.Arg336Trp		
		p.Thr360Asn		
		p.Pro448Ser		
		p.Ala186Thr		
		p.Asn6Asp		
		p.Glu190fs		
***TSC2***	16p13.3	p.Lys533Gln	Tuberous sclerosis	
		p.Ala678Thr		
		p.Glu984Gln		
		p.Ala1097Val		
		p.Ser1698Arg		
		p.Ile64Val		
		p.His152Asp		
		p.Lys533Gln		
		p.Lys954Arg		
		p.Glu984Gln		
		p.Ala1429Ser		
		p.Glu234fs		
		p.Val1034Ile		
		p.Asp647Asn		
		p.Val296Met		
		p.Leu361Val		
		p.Arg622Trp		
***RELN***	7q22.1	p.Gly370Arg		
		p.Asn1159Lys		
		p.Ser1719Leu		
		p.Arg1742Gln		
		p.Val1762Ile		
		p.Arg2290His		
		p.Thr2718Ala		
		p.Gly1280Glu		
		p.Arg255Trp		
		p.Arg2292Cys		
		p.Thr1904Met		
		p.Arg1727Trp		
		p.Pro1580Leu		
		p.Pro3379Arg		
		p.Ile3374Val		
		p.Pro2245Arg		
		p.Leu2057Val		
		p.Arg822Gly		
		p.Arg2242Ser		
		p.Leu522Phe		
		p.Arg3439Gln		
		p.Val3426Ile		
		p.Asn2535Lys		
		p.Val2372Met		
		p.Asp2309Asn		
		p.Asp2309Asn		
		p.Arg1742Gln		
		p.Glu1410Lys		
		p.Leu411Ile		
		p.Val3426Ile		
		p.Lys751Thr		
		p.Tyr1183Cys		
		p.Pro638Leu		
		p.Ala3327Ser		
		p.Pro638Leu		
		p.Arg1727Gln		
		p.Ser142Asn		
		p.Ala3281GlnfsTer11		
		p.Gly1113Glu		
		p.Tyr2154Cys		
		p.Gly2802Arg		
		p.Gly2783Cys		
		p.His798Asn		
		p.Pro844Leu		
		p.Asp763Gly		
		p.Pro672Leu		
		p.Tyr723Cys		
		p.Ser2513Cys		
		p.Ala2545Val		
		p.Trp1083fs		
		p.Pro1340Leu		
		p.Asp2309Asn		
		p.Gln3313Arg		
***EN2***	7q36.3	p.Pro142Arg		*EN2*-KO
***PTEN***	10q23.31	p.Tyr178Ter		
		p.Asp22Glu		
		p.Gln214Ter		
		p.Met134Ile		
		p.Arg130Ter		
		p.Leu139Ter		
		p.Arg173His		
		p.Trp274Leu		
		p.Met134Thr		
		P.Leu182Ser		
		p.Ile50Thr		
		p.Val133Ile		
		p.Lys164Asn		
		p.Ser59Ter		
		p.Arg233Ter		
		p.Ser170Arg		
		p.Gly136fs		
		p.Arg154fs		
***UBE3A***	15q11.2	p.Ala201Thr	Angelman	
		p.Ala178Thr		
		p.Ala198Thr		
		p.192_193del		
		p.Arg506Cys		
		p.Ala599fs		

**Table 2:** MiRNAs dysregulated in ASD and identified in animal models.

miRNA	Function	Reference	Models
miRNA-125b	miRNA-125b regulates the expression of *FMR1*; it is upregulated with the *FMR1* Knock-out. It contributes to the alteration of synaptic plasticity as in FXS.	[55]	KO mice
miRNA-132	miRNA-132 and miRNA-125b are involved in synaptic plasticity. Furthermore, miRNA-132 targets the 3’UTR sequence of *MECP2* and prevents its expression. As a result of the pain, it is downregulated, inducing an increase in the analgesic response and in the expression of *MECP2*. Furthermore, it is involved through the p-CREB/miR-132 pathway in the regulation of expression between *PTEN* and *MECP2*.	[55,60,61]	KO mice
miRNA-146a, miRNA-146b	miRNA-146a and miRNA-146b were observed in the RTT syndrome. A study on the *MECP2*-KO mice model observed that they are downregulated by inducing overexpression of the *IRAK1* gene which leads to an inflammatory state of the brain tissue such as in ASD.	[57,58,59]	KO mice
miRNA-137	When miRNA-137 is downregulated, it influences the expression of many genes implicated in neurodevelopment. This means they lead to learning and memory deficits, and they are therefore associated with ASD. Furthermore, miRNA-137 is associated with *SHANK2*, as both are expressed in synapses. miRNA-137 targets the 3’UTR sequence of *SHANK2* so as to repress the expression of the SHANK2 protein.	[61,62,63]	KO mice
miRNA-134	The KO of *UBE3A* allowed observing an upregulation of miRNA-134 by belonging to the miRNA379-410 cluster that regulates synaptic plasticity. Furthermore, the UBE3A1 RNA transcript acts as a rival endogenous dendritic RNA for miRNA379-410.	[65]	KO mice
miRNA-134-5p	miRNA-134-5p is overexpressed in the brains of patients with ASD; it controls the development of the spine.	[66,67]	VPA rats
miRNA-138-5p	miRNA138-5p is implicated in the development changes of dendritic spines and in the narrowing of the spine.	[66,67]	VPA rats
miRNA-34a	VPA regulates miRNA-34a levels which in turn regulate *BCL2* expression. In the cerebral cortex of VPA rats immediately after birth, the upregulation of miRNA-34a and a reduction in *BCL2* expression were observed.	[68]	VPA rats
miRNA-181c	miRNA-181c is upregulated in the amygdala of ASD patients; its function is associated with the development of the nervous system.	[69,70,71]	VPA rats
miRNA-30d	miRNA-30d is also upregulated in VPA rats, and its function is associated with the development of the nervous system.	[69,70,71]	VPA rats
